# Therapeutic inhibition of proteasomes in systemic lupus erythematosus

**DOI:** 10.1186/1479-5876-9-S2-I9

**Published:** 2011-11-23

**Authors:** Falk Hiepe, Bimba Hoyer, Tobias Alexander, Adriano Taddeo, Reinhard Voll, Andreas Radbruch

**Affiliations:** 1 Clinic for Rheumatology and Clinical Immunology, Charité – Universitätsmedizin Berlin, Germany; 2 Deutsches Rheumaforschungszentrum Berlin, a Leibniz Institute, Berlin, Germany; 3 Clinic for Rheumatology and Clinical Immunology, University Hospital, Freiburg, Germany

## 

When activated, B cells can differentiate either into memory B cells, or into plasma cells, which secrete the B cells antibody at high rates. Plasma cells can be short-lived or long-lived. Long-lived plasma cells are maintained mostly in the bone marrow, in privileged survival niches, which are organised by dedicated stroma cells [1]. Long-lived plasma cells constitute a discrete type of immunological memory cells, providing humoral protection to recurrent pathogens. In immune-mediated diseases, like allergy or inflammatory rheumatic diseases, however, memory plasma cells can turn detrimental, constantly providing pathogenic antibodies. In systemic lupus erythematosus (SLE), autoreactive memory plasma cells, secreting antibodies against self antigens such as DNA, are readily detectable. These cells represent a therapeutic challenge. While short-lived plasma cells, respectable their precursors and their generation, are targeted by conventional immunosuppression with drugs such as cyclophosphamide, memory plasma cells do not respond, continue to secrete antibodies, and thus sustain persistence of disease [2,3]. New therapies, targeting memory plasma cells, have to be developed, to meet this challenge. Several different therapeutic strategies have been proposed so far.One of the more promising strategies aims at the inhibition of proteasomes, since this strategy has been shown to be effective to eliminate multiple myeloma cells, i.e. transformed plasma cells. In a first study, Neubert and collegues could show in 2008 that in the NZB/W mouse model of SLE, memory plasma cells are depleted from spleen and bone marrow by the proteasome inhibitor bortezomib (Velcade) [4]. Clinically, treated mice showed reduced nephritis and prolonged survival.

Consequently, the groups of ReinhardVoll and our group initiated a clinical trial of bortezomib for the treatment of patients with SLE. Those patients had not responded to other treatments earlier and showed persistent autoantibody titers, indicating the presence of autoreactive memory plasma cells. Four patients with active SLE, previously treated with cyclophosphamide, received bortezomib (Velcade®) at doses of 1.3mg/m^2^ on days +1, 4, 8, 11 followed by a 10 days treatment-free interval for four cycles along with 20mg dexamethasone according to the approved myeloma protocol. Under proteasome inhibition, a significant clinical improvement was achieved in all patients associated with a reduction in lupus-specific autoantibodies. In addition, protective, vaccine-specific antibodies and total immunoglobulin levels dropped significantly, suggesting a depletion of bone marrow memory plasma cells secreting these antibodies.In all patients, like in the NZB/W mice, repeated treatment was needed for sustained efficacy, indicating that autoreactive memory plasma cells are continuously generated in chronic disease. Thus, a therapeutic regimen combining B cell depletion with memory plasma cells depletion might be preferable. First experiments in NZB/W mice showed a sustained effect of combination therapies depleting plasma cell precursors as well as fully differentiated plasma cells.

In conclusion proteasome inhibition in SLE has demonstrated the therapeutic relevance of autoreactive memory plasma cells as such, and it constitutes a new therapeutic option, which has to be developed with respect to sustained effectivity, minimization of side effects and reduction of risk of infection.
